# Application of Advanced Platelet-Rich Fibrin in Oral and Maxillo-Facial Surgery: A Systematic Review

**DOI:** 10.3390/jfb15120377

**Published:** 2024-12-14

**Authors:** Marek Chmielewski, Andrea Pilloni, Paulina Adamska

**Affiliations:** 1 Private Dental Practice, 14 Kolberga Street, 81-881 Sopot, Poland; machmielewski@proton.me; 2 Section of Periodontics, Department of Oral and Maxillo-Facial Sciences, Sapienza University of Rome, 00-185 Rome, Italy; andrea.pilloni@uniroma1.it; 3 Division of Oral Surgery, Medical University of Gdańsk, 7 Dębinki Street, 80-211 Gdańsk, Poland

**Keywords:** advanced platelet-rich fibrin, A-PRF, autografts, dentistry, growth factors, wound healing

## Abstract

**Background:** Advanced platelet-rich fibrin (A-PRF) is produced by centrifuging the patient’s blood in vacuum tubes for 14 min at 1500 rpm. The most important component of A-PRF is the platelets, which release growth factors from their ⍺-granules during the clotting process. This process is believed to be the main source of growth factors. The aim of this paper was to systematically review the literature and to summarize the role of A-PRF in oral and maxillo-facial surgery. **Materials and Methods:** A systematic review was carried out, following the Preferred Reporting Items for Systematic Reviews and Meta-Analyses (PRISMA) guidelines (PROSPERO: CRD42024584161). **Results:** Thirty-eight articles published before 11 November 2024 were included in the systematic review. The largest study group consisted of 102 patients, and the smallest study group consisted of 10 patients. A-PRF was most often analyzed compared to leukocyte-PRF (L-PRF) or blood cloth. A-PRF was correlated with lower postoperative pain. Also, A-PRF was highlighted to have a positive effect on grafting material integration. A-PRF protected areas after free gingival graft very well, promoted more efficient epithelialization of donor sites and enhanced wound healing. **Conclusions:** Due to its biological properties, A-PRF could be considered a reliable addition to the surgical protocols, both alone and as an additive to bio-materials, with the advantages of healing improvement, pain relief, soft tissue management and bone preservation, as well as graft integration. However, to determine the long-term clinical implications and recommendations for clinical practice, more well-designed randomized clinical trials are needed in each application, especially those with larger patient cohorts, as well as additional blinding of personnel and long follow-up periods.

## 1. Introduction

Since the first clinical introduction of platelet-rich fibrin (PRF) in dentistry by Choukroun in 2001 [1,2,3,4], PRF has grown to be one of the most influential natural resources in regenerative dentistry. In 2014, advanced platelet-rich fibrin (A-PRF) was introduced and described by Ghanaati et al. [5] and Choukroun [6] as one of the most promising iterations.

So far, three generations of blood-derived platelet-rich preparations rich in growth factors have been identified:

I. platelet-rich plasma (PRP), plasma-rich in growth factors (PRGF);
II. platelet-rich fibrin;
III. advanced platelet-rich fibrin, advanced platelet-rich fibrin plus (A-PRF+), injectable platelet-rich fibrin (I-PRF), concentrated growth factor (CGF), titanium platelet-rich fibrin (T-PRF) and autologous fibrin glue (AFG) [7,8].

**Figure 1:**
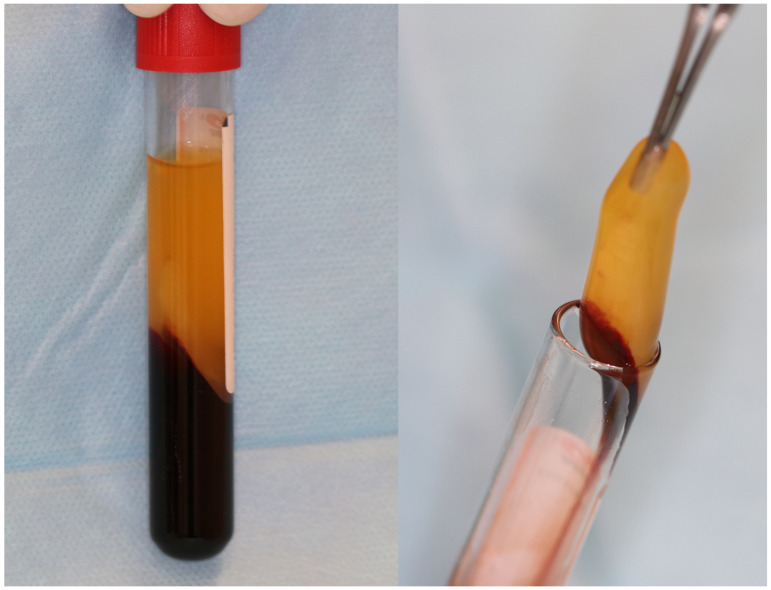
A-PRF clot in glass-coated plastic tubes.

A-PRF (Figure 1) is derived from the patient’s venous blood drawn prior to surgery (most commonly from the brachial vein) without the use of anticoagulants. The autogenous origin ensures no undesirable antigen reactions after graft placement and during integration. The gelatinous consistency and state are similar to PRF due to a similar process of obtaining it [9,10]. It consists of a fibrinous matrix with mixed platelets, leukocytes, macrophages, neutrophils, proteins, cytokines and growth factors present in the blood, which are densely trapped. The matrix ensures mechanical strength and serves as a binding agent for cytokines and growth factors. A looser structure provides a more even distribution of cytokines than PRF, and more interfibrous space allows for more cells present in the cloth [5,9,10].

Cytokines and growth factors play an important role in biochemical properties. A-PRF affects both soft and hard tissues, mainly due to the effects on tissue fibroblast regeneration [10,11] and osteoblasts [11]. The most important part of A-PRF are platelets that release growth factors from their ⍺-granularities during clotting. This process is believed to be the main source of growth factors, such as platelet-derived growth factor (PDGF), vascular endothelial growth factor (VEGF), fibroblast growth factor (FGF), transforming growth factors (TGF-⍺ and β), bone morphogenetic proteins (BMPs, such as BMP-2) and matrix metalloproteinases (MMPs, such as MMP-9). A-PRF has been shown to release more of these growth factors and MMPs than PRF due to its modified centrifugal processing. Research works indicate that A-PRF is currently the most favorable PRF-like material available, providing cytokines for up to 10 days [6,9,12,13,14]. Also, the addition of Ca^2+^ can alter the amount of growth factors secreted into surrounding tissues [15].

In order to obtain A-PRF, the patient’s blood has to be placed in a centrifuge and processed at 1500 rpm for 14 min, as opposed to the classical PRF at 3000 rpm for 12 min. There is also no anticoagulant, which gives A-PRF better biological properties compared to classical PRF. The lower centrifugation parameters of A-PRF compared to PRF or L-PRF do not allow the platelets to be pushed down the tube. The advantage of lower centrifuge speed in A-PRF preparation is the improvement of properties. Neutrophils can migrate to the fibrin matrix [6]. It is proven that in A-PRF in the peripherals of the cloth, there are platelets present. The difference in the processing can be responsible for more optimized, longer lasting and more even distribution and release of growth factors from A-PRF to the surrounding tissues, affecting tissue regeneration and maturation in comparison to PRF and L-PRF. The distribution of lymphocytes, macrophages and stem cells is greater in the proximal part of the cloth, whereas neutrophils are located mainly in the distal part [2,5,6]. It is postulated to reduce the formation time of A-PRF to 8 min, which further improves its biological properties. Further modifications of centrifugation led to the creation of I-PRF (700 rpm, 3 min), which has even more concentrated factors than advanced and leukocyte PRF. In comparison to PRF, it must be used within 20 min of preparation vs. 4 h for A-PRF. It is very important to maintain the speed of rotations per minute, time, relative centrifugal force, diameter and angulation of the rotor in the centrifuge, size and type of test tubes. Any change may lead to the incorrect production of blood-derived platelet-rich preparations rich in growth factors and loss of the related properties. Horizontal vs. fixed angle centrifugation is also important because horizontal centrifugation leads to four times higher cell concentration, which is evenly distributed across the top of the tube and is not damaged as much. The more hydrophilic the tube surface, the better the clot quality [2,5,6,16,17].

A-PRF is primarily used in surgical procedures. However, it can also be used in general oral surgery (filling the post-extraction socket, in treatment of alveolar osteitis, used to control bleeding–hemostatic effect), endodontics (in regenerative endodontic treatment (RET) of traumatized immature non-vital teeth), implantology (bone regeneration, socket preservation, alveolar ridge preservation, maxillary sinus augmentation), periodontics (in treatment recessions) and to enhance general wound healing (reduced pain, swelling or trismus) [2,18,19,20,21,22,23,24,25,26,27,28,29,30,31,32,33,34,35,36,37,38,39,40]. This makes A-PRF just as versatile as PRF. The aim of this paper was to collect and review the information about A-PRF, its role and its advantages in oral and maxillo-facial surgery.

## 2. Materials and Methods

### 2.1. Focused Question

The following focused question was defined: ‘Does A-PRF provide better clinical outcomes than other materials used in exact oral and maxillo-facial procedures?’ The PICO (population, intervention, comparison, outcome) was used:

P—at least 10 people qualified for the use of A-PRF;

I—dental procedures (i.e., tissue repair, socket management, sinus lifts) combined with the use of A-PRF as sole/combined bio-material;

C—defined approaches using A-PRF only or with other PRF types and other conventional methods;

O—soft and/or hard tissue reconstruction of the periodontium, alveolar bone or tooth structure.

### 2.2. Search Strategy

The search was conducted using PubMed, Scopus and Google Scholar web databases. The PRISMA guidelines (Preferred Reporting Items for Systematic Reviews and Meta-Analyses) were used [41]. The study protocol was registered with the PROSPERO (Prospective Register of Systematic Reviews, CRD42024584161). The search terms used were ‘advanced platelet-rich fibrin’, ‘A-PRF’, ‘dentistry’, ‘oral surgery’ and ‘maxillo-facial surgery’. The last manuscript search was conducted on 11 November 2024.

### 2.3. Selection Criteria

#### 2.3.1. Inclusion Criteria

For the use of A-PRF in dentistry, only randomized clinical trials were used, with a minimum of 10 patients treated. Studies were selected from the databases according to the following criteria: (1) only human studies, (2) studies regarding A-PRF use in oral and maxillo-facial procedures, (3) studies carried out and published from the beginning of 2014 up to 11 November 2024, (4) studies published in English, (5) randomized clinical studies on a group of at least 10 people.

#### 2.3.2. Exclusion Criteria

The exclusion criteria were (1) studies conducted on animals or in vitro, (2) studies not available in full text, (3) group of patients less than 10, (4) studies written in language other than English.

### 2.4. Study Selection and Data Extraction

The retrieved publications were initially scanned in accordance with the selection criteria. Only publications fulfilling the inclusion criteria were taken into account. Duplicates from the databases were discarded. Upon screening the abstracts, selected articles were obtained in full text. If the abstract screening and title did not provide enough decisive information to include the article, further screening of the whole publication was carried out. Lastly, the full-text manuscripts were reviewed according to the selection criteria. Subsequently, the first author analyzed the publications and critically assessed the articles. In cases of uncertainty, the analysis was completed by a third author.

The data extracted included general publication characteristics (authors, year of publication, journal), case type, number of patients, outcome and complications. The analyzed data from the publications were divided according to the procedure and presented in tables for comparison.

### 2.5. Quality Assessment

In this article, the risk of bias was assessed using the Revised Cochrane risk of bias tool for randomized trials (RoB 2) [42,43]. These procedures were performed by the third and first authors. In case of disagreement, a consensus reading was made.

### 2.6. Statistical Analysis

Statistical analysis was performed using STATISTICA 13.3 (TIBCO, Palo Alto, CA, USA) licensed to the Medical University of Gdańsk. The number of studies, included publications and patients studied were quantitatively summarized.

A qualitative synthesis was conducted using the established criteria, summarizing the available research and analyzing the structure of PICO; the advantages, disadvantages, future research directions and the relationship with previous scientific reports were discussed. Quantitative analysis (meta-analysis) was not performed due to the heterogeneity of studies.

## 3. Results

### 3.1. Search Outcomes

**Figure 2:**
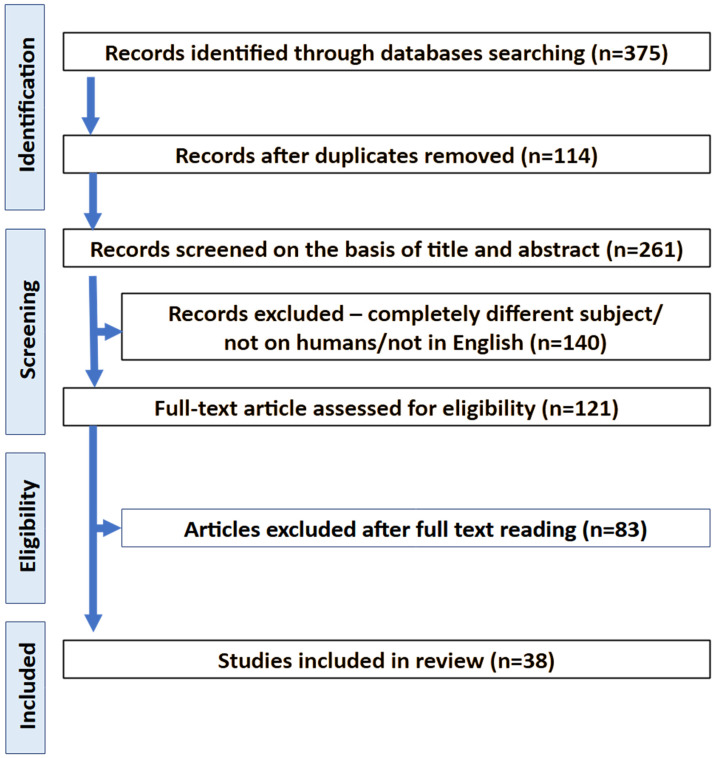
PRISMA workflow.

After eliminating duplicates, 375 articles remained to be reviewed. After exclusion of duplicates, 261 articles remained. The screening of titles and abstracts excluded 140 articles. Ultimately, 38 articles were selected for systematic review. They were further divided into their respective category depending on the procedure carried out in the study (Figure 2). The first identified studies are from 2015. All randomized clinical studies are depicted in Table 1. The total number of patients analyzed in all studies was 1307 [2,19,20,21,22,23,24,25,26,27,28,29,30,31,32,33,34,35,36,37,38,39,40,44,45,46,47,48,49,50,51,52,53,54,55,56,57,58,59,60].

### 3.2. Results of Individual Studies

#### 3.2.1. A-PRF in Alveolar Ridge Preservation After Tooth Extraction

A-PRF is a material densely packed with growth factors and cytokines. This makes it a promising material for ridge preservation after tooth extraction. To date, there have been four randomized clinical trials (RCTs) that used A-PRF, attempting to hinder bone resorption after tooth extraction [30,32,39,48].

Most often, A-PRF is compared to L-PRF or blood cloth. A radiological analysis performed by Castro et al. [30] showed that A-PRF presented better results than blood cloth alone in all analyzed points (horizontal, buccal, palatal resorptions, ridge width changes, vertical resorption and socket fill) but fared very similar to L-PRF in terms of alveolar ridge preservation. A morphometric bone analysis (histological, 2D and 3D micro-computed tomography (micro-CT)) presented similar results between the study groups, with both outperforming the blood cloth control group. Although both PRFs hindered bone resorption, they could not counteract it completely.

Two randomized controlled trials (RCTs) conducted by Clark et al. [32] and Ivanova et al. [39] also include the analysis of A-PRF behavior when combined with FDBA in alveolar ridge preservation.

In the operating procedure described by Clark [32], mucoperiosteal flap elevation was not performed; single-root teeth were extracted; and non-steroidal anti-inflammatory drugs (NSAIDs; 600 mg ibuprofen) and a 0.12% chlorhexidine mouth rinse were administered at the beginning of the surgery. Conversely, Ivanova [36] elevated the flap from both the palatal and buccal sides, extracted multiple-root teeth and administered a 0.12% mouth rinse twice daily for a period of two weeks following the surgical procedure. Additionally, they administered an antibiotic (1000 mg amoxicillin) and a non-steroidal anti-inflammatory drug (nimesulide 100 mg).

For the purpose of measuring the dimensions of the operating area, Ivanova et al. [39] employed the use of a Trios intraoral scanner for the analysis of a virtual model, whereas Clark et al. [32] utilized a more conventional methodology, namely the fabrication of a stent from light-cured resin derived from an alginate impression, which was then measured with a periodontal probe for height and calipers for width. In both cases, a trepanning burr was used to harvest the bone core. The primary outcome was assessed in both randomized controlled trials (RCTs) by means of a comparison of the vertical and horizontal dimensions of the alveolar ridge, while the secondary and tertiary outcomes were evaluated through histomorphometric and micro-computed tomography (micro-CT) analyses.

Clark et al. [32] observed that the groups using A-PRF and FDBA demonstrated lower alveolar ridge height reduction compared to the blood cloth group. Histomorphometric analysis showed that A-PRF and FDBA + A-PRF allowed the formation of a denser trabecular structure. In terms of augmenting material integration, A-PRF enhanced the integration of FDBA by decreasing the amount of residual graft material and showed significantly higher maturation of the structure compared to FDBA alone. Bone vitality was greatest in the A-PRF group, but mineral bone density was best in the FDBA group; both results showed statistical significance. The study conducted by Ivanova et al. [23] presented similar results regarding the superiority of A-PRF and FDBA over blood cloth regarding alveolar ridge dimension preservation, but in the case of vital bone formation, A-PRF did not perform better than FDBA.

In contrast, Pereira et al. investigated the effect of A-PRF on socket healing after extraction of upper wisdom teeth. A clinical and cone-beam computed tomography (CBCT) study was conducted, and no significant benefit was found between A-PRF application and treatment without the use of growth factors [48].

#### 3.2.2. A-PRF Effect on Postoperative Pain, Swelling and Trismus

A meta-analysis of nine studies revealed that A-PRF administration was demonstrated to effectively reduce postoperative pain in seven studies [19,24,31,37,46,49,51,56,60].

Caymaz et al. [31] compared A-PRF to L-PRF in terms of pain, swelling and trismus after third molar extractions. Apart from the VAS pain score, in the study, pain was also measured by analgesic drug usage. The most noticeable difference was visible in the first 3 postoperative days. Compared with L-PRF, A-PRF presented much lower VAS pain scores in the first 3 postoperative days, which slowly equaled the L-PRF group by day 7. The difference in the number of analgesics used by the A-PRF group was noticeably lower from days 2 to 6. In terms of swelling and trismus, there were no significant differences between the two groups.

Zahid and Nadershah [60] performed third molar extractions with the use of A-PRF as a test group or with blood cloth only as a control group. The A-PRF group showed decreased pain and swelling compared to the control group over 7 postoperative day periods. Moreover, the healing aspects were checked (pocket depth, gingival recession, clinical attachment level), but no significant advantages were detected compared to the A-PRF group.

In the study by Nowak et al. [46], the impact of A-PRF application on the surgical removal of third molars was examined, with a particular emphasis on its influence on healing and the concentration of C-reactive protein. A more rapid decline in C-reactive protein levels was observed in subjects who underwent third molar extraction and subsequently utilized A-PRF. The application of A-PRF resulted in accelerated healing and a reduction in the incidence of alveolar osteitis.

Starzyńska et al. [19] assessed the influence of A-PRF on selected clinical features following surgical removal of the impacted mandibular third molars. A-PRF reduced the pain intensity, analgesic intake, trismus, edema, the presence of hematomas and skin warmth.

In contrast to the studies above, Torul et al. [56] did not detect significant advantages of using A-PRF in the procedure of lower third molar extraction. The control group (blood cloth) was compared with CGF and A-PRF groups in terms of pain, swelling and trismus. The only significant result regarding A-PRF usage was detected in terms of swelling on the seventh postoperative day compared to the CGF group (the Tragus–Pogonion measurement). In all categories, A-PRF exhibited a similar outcome to that of the control group, demonstrating minimal improvement. Additionally, Praganta et al. [49] did not identify a notable distinction in the reduction in pain and swelling between the A-PRF and gelatin sponge groups following the extraction of wisdom teeth.

The study conducted by Hartlev et al. [37] researched A-PRF usability in pain management in the surgical procedure of lateral ridge augmentation following mandibular ramus block harvesting. The test group, which utilized A-PRF, demonstrated superior results in terms of VAS pain scoring compared to the control group, which employed a blood cloth. Although throughout the seven measured days, pain decrease was only significant in the first postoperative day, the A-PRF group showed lowered VAS pain score up to the fifth day. Taking into account the fact that the pain associated with the surgical protocol used is usually low according to the authors, decreasing the pain levels further can still improve the quality of life of patients.

In the case of the treatment of recession with the pin hole method and A-PRF simultaneously in the study by Al-Barakani et al. [24], a reduction in the level of postoperative pain was observed.

In the study by Şen DÖ et al. [51], the effects of L-PRF and A-PRF as a palatal bandage following free gingival graft on patients’ morbidity and oral-health-related quality of life were examined. The control group without growth factors had higher OHIP-14 total scores than the other groups. The PRF groups showed an improvement in the quality of life and took less painkillers.

#### 3.2.3. A-PRF Use in Implantology

The field of implantology is dedicated to identifying the most effective solutions and alternatives to address the challenges encountered by patients. A review of the literature revealed three randomized controlled trials investigating the impact of A-PRFs in surgical protocols.

The studies conducted by Kalash et al. [44] and Alhaj et al. [25] focused on the effect of A-PRF in immediate implantation when combined with grafting material. Both pieces of research indicated that A-PRF addition improved the clinical outcomes. The probing depth and implant stability checked by Kalash et al. [44] outperformed the control group, showing fewer variations in the results at follow-up points. The addition of A-PRF to the surgical protocol was found to enhance the marginal bone height and bone density in both studies. The study by Alhaj et al. [25] demonstrated a statistically significant difference between the groups at the final follow-up.

The third study concentrated on the comparison of A-PRF and FGG, with an evaluation of their potential for improving the keratinized tissue around implants. Alsahli et al. [26] found that there were no significant differences between the two groups, which indicated that A-PRF performed similarly to FGG. The use of A-PRF was shown to lower the postoperative pain up to the sixth day. In consideration of the absence of a second surgical site, A-PRF emerges as a compelling alternative to the established protocol. The drawback is that the thickness of the keratinized tissue in the A-PRF group showed a gradual decrease over time.

#### 3.2.4. A-PRF in Hard Tissue Healing

To date, six randomized controlled studies have been conducted on the use of A-PRF alone [27,38,58] or in conjunction with other PRF types [33,45] or membranes [50]. A-PRF was highlighted to have a positive effect on grafting material integration in three of these studies [35,37,38]. Furthermore, the A-PRF groups indicated enhanced biometric bone quality with lower grafting material resorption. This allows for maintaining the alveolar ridge dimensions more predictably, which leads to more predictable future implant placements. A study by Dayashankara Rao et al. [33] also focused on the periodontal status and pocket depth in recipient places. The test group performed better than the control, showing improvement in both aspects with lower mobility scores. Yewale et al. [51] checked the pain and swelling of the test and control groups. While the pain levels remained similar between the two groups, the swelling decreased noticeably in the test group, improving patient postoperative comfort. Angelo et al. [27] used A-PRF as the membrane for a bio-material in piezotome-enhanced subperiosteal tunnel technique (PeSPTT). The results demonstrated that the use of A-PRF resulted in more consistent outcomes, with enhanced bone formation quality for implant placement.

However, the study conducted by Hartlev et al. [38] presents a contrasting approach, directly comparing A-PRF membranes covering autograft material with autografts combined with DBBM and a collagen membrane. The test and control groups showed no significant differences in terms of vital and non-vital bone formation, the amount of new blood vessels formed and the improvement in soft tissues.

In a previous study, Lavagen and colleagues [45] demonstrated the efficacy of A-PRF in the treatment of alveolar clefts with iliac bone grafts. In the study, the authors evaluated the efficiency of using A-PRF by comparing the volumes of newly formed bone after a bone graft combining autogenous iliac crest bone with either PRF or A-PRF. In groups with A-PRF placement, bone regeneration was more effective.

In the study conducted by Rachna et al. [50], the impact of applying A-PRF or A-PRF combined with eggshell membrane following tooth extraction was investigated. In the A-PRF and eggshell membrane group, after 3 and 6 months, the bone density in the CBCT scan was higher than in the A-PRF group.

#### 3.2.5. A-PRF in Soft Tissue Healing

The present study analyzed seven studies [24,28,29,40,44,47,53] that were based on research on palatal FGG surgical procedures. A-PRF was made the test group for patching the donor site. The clinical outcomes take into account direct soft tissue changes (color changes, contour changes, texture, epithelialization, wound area reduction) and postoperative pain (VAS score). In terms of color, contour and texture, in the controlled trial conducted by Bahmanamm [28], A-PRF showed better results than the control group. The wound margin analysis showed a consistently better score for the A-PRF group. Moreover, A-PRF promoted more efficient epithelialization of donor sites and enhanced wound healing. The effect was most profound from day 7 to 30. In that period, the percentage wound area reduction in the study by Sousa et al. [53] showed a statistically significant (7th and 30th day) or close to significant (14th day) result. Regarding pain perception, the VAS score measured in both studies [40,44] showed reductions in the A-PRF group, with statistical significance in Bahmanamm’s trial [28].

In their study, Öngöz Dede et al. [47] analyzed the effects of concentrated growth factors in combination with A-PRF used during coronally advanced flap in the treatment of multiple gingival recessions (type 1 recessions). In the case of using platelet-rich preparations in the treatment of recession, significant improvements were determined in the clinical attachment level, vertical gingival recession, horizontal gingival recession, gingival thickness, width of keratinized gingiva, percentages of the mean and complete root coverage compared to a control group that did not receive CGF and A-PRF.

In the study conducted by Al-Barakani et al. [24], the treatment of recession (types I and II, as defined by the Miller classification) using A-PRF in the pinhole surgical technique (PST) was demonstrated to be more effective than the application of a resorbable collagen membrane (RCM) in PST. However, both methods were ultimately deemed unsatisfactory.

In a further study, Barakat et al. [29] examined the clinical effects of A-PRF and connective tissue grafts (CTGs) in the treatment of gingival black triangle (GBT) using Han and Takei’s method. The results of the study indicated that both A-PRF and CTG yielded comparable outcomes when used in conjunction with Han and Takei’s techniques.

#### 3.2.6. A-PRF Effect on Hemostasis

The studies conducted by Brancaccio et al. [2] and Giudice et al. [33] gathered a total of 142 patients taking antiplatelet drugs. Both studies used the direct comparison of blood cloth (control), hemostatic plug (HEM), A-PRF and L-PRF (test groups). Each patient had four non-adjacent teeth removed, without flap elevation. Based on the trials, A-PRF exerted the best hemostatic effect. When postoperative bleeding was assessed 30 min after the extractions, A-PRF demonstrated superior performance to the control group, with statistical significance [37]. Branaccio et al. [2] obtained similar results on the broader patient groups but with both A-PRF and L-PRF significantly outperforming the control group. Furthermore, A-PRF showed statistical significance in bleeding reduction compared to HEM. Further evaluation of wound healing showed that A-PRF and L-PRF promoted wound closure more effectively than HEM and the control in both studies [2,36]. Regarding patient preference, Giudice et al. [36] surveyed patients one and two weeks after the extractions. After one week, most patients selected A-PRF, with control sites being the second choice. On the second follow-up, A-PRF and L-PRF were ex aequo the most often selected options.

#### 3.2.7. A-PRF in Maxillary Sinus Augmentation

There have been two randomized controlled trials identified regarding A-PRF use in maxillary sinus lift. Trimmel et al. [57] followed the augmentation by placing the implants in the augmented sites, measuring the implant stability quotient (ISQ) throughout the osseointegration process. The control group showed a significantly better ISQ at weeks 6 and 8. During implant placement, bone samples were collected and analyzed. The micromorphometry results favored the A-PRF group, as it presented significantly better results in terms of bone surface/bone volume ratio, bone surface density, trabecular thickness and connectivity. Histomorphometry using hematoxylin–eosin staining did not present a difference between the two groups.

In contrast, Dragonas et al. [34] compared A-PRF and plasma rich in growth factors combined with deproteinized bovine bone mineral during sinus lift augmentation. The mean percentage of mineralized bone after the healing period was higher in the group with growth factors, but there was no statistical significance in samples without growth factors. Adding A-PRF and PRGF to DBBM did not improve new bone formation in the sinus lift procedure. Neither platelet-rich preparation was better than the other in any of the parameters studied.

#### 3.2.8. A-PRF in Intrabony Defect Management

Additionally, A-PRF is emerging as a material with valuable applications in periodontology. The study by Ghonima et al. [35] compared A-PRF + BCP with BCP + saline. The test group did not show statistically significant improvements over the control group, though it must be noted that the outcomes were slightly more favorable toward A-PRF in terms of PD reduction and CAL gain. Both groups also showed a significant reduction in the plaque index at 9-month follow-up. On the other hand, Csifó-Nagy et al. [21] compared A-PRF with EMD. The A-PRF test group performed similarly to the EMD group, with no statistical differences in terms of PD, GR, CAL and bone sounding changes. Moreover, both materials used in the procedure lowered the full mouth bleeding score at a 6-month follow-up.

#### 3.2.9. A-PRF Use in Alveolar Osteitis (Dry Socket)

The research conducted by Yüce et al. [59] focused on the pain levels and soft and hard tissue differences in patients suffering from alveolar osteitis. The debridement and irrigation were accompanied by A-PRF in the test group. In terms of pain reduction, researchers found that the pain decreased rapidly and continually on days 1, 3, 5 and 7 in conjunction with analgesic usage. Compared with control, the results were statistically significant. Looking into soft tissue changes, the test group presented significantly higher epithelium healing rates at all times. The degree of hard tissue healing was estimated by calculating the gray level pixel count. The measurements taken at second and third months showed statistical significance in average pixel values for the test group.

#### 3.2.10. A-PRF in Endodontic Surgery

There have been two studies that examined A-PRF usage in endodontics [40,50]. A-PRF was introduced to the apical root resection protocol with paramarginal mucoperiosteal flap release. Patients were only selected if they required endodontic treatment of the maxillary second premolar and had lesions measuring 6–12 mm (cone-beam computed tomography measurement). In terms of pain reduction, A-PRF lowered the VAS score, but with no statistical significance. The same was true for analgesic usage. A-PRF also fared better than control in mouth opening and chewing functions, sleep impairment, inflammation of the operation site and discomfort in that area [50].

In the study by Jayadevan et al. [40], A-PRF and PRF were used as a scaffold in the regenerative endodontic treatment (RET) of traumatized immature non-vital teeth. Immature teeth have narrow canal walls. A-PRF yielded higher root dentin thickness than PRF.

#### 3.2.11. A-PRF in Treatment of Oroantral Communication

In the study by Śmieszek-Wilczewska et al. [54], oroantral communication treated with A-PRF resulted in fewer complications and less pain. In addition, the use of A-PRF alone as clots resulted in complete healing and closure of the connection. Additional methods, such as regional flaps, were not necessary.

### 3.3. Quality Assessment

**Figure 3:**
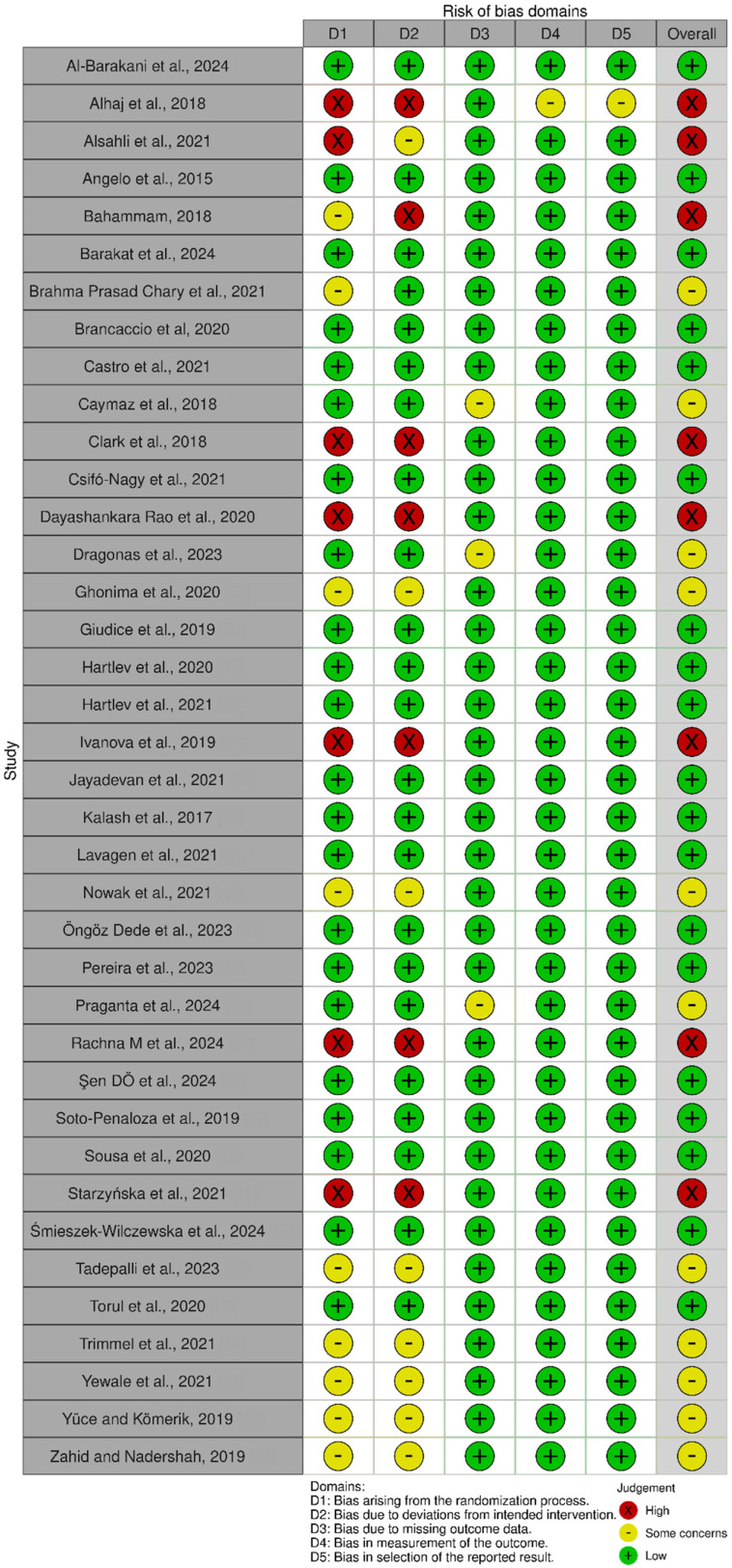
The risk of bias assessment using RoS 2 [2,19,20,21,24,25,26,27,28,29,30,31,32,33,34,35,36,37,38,39,40,44,45,46,47,48,49,50,51,52,53,54,55,56,57,58,59,60].

The risk of bias assessment using RoS 2 is described in Figure 3.

## 4. Discussion

A-PRF is still a novel material, related to the original PRF; nonetheless, further research must be conducted on its applications. The existing randomized controlled trials proved to be a scarce source of data regarding the sole use of A-PRF in dental surgical procedures. The variety of different combinations of bio-materials used with advanced platelet-rich fibrin often led to slightly altered results between the trials regarding specific usage. The vast majority of studies limited themselves to small patient cohorts, rarely exceeding 40 patients in the test groups. The follow-up periods were also a variable factor between the studies, depending mainly on the procedure performed but also on the protocol followed by the surgeons. In the presented studies, the cohorts were divided mostly equally, with few exceptions [31,32,37,38,53]. Despite these differences, the results demonstrated that A-PRF has a vast spectrum of applications and provides a valuable supplementary benefit when integrated into the surgical protocol. Studies that employed A-PRF as the sole comparison standard primarily investigated its efficacy in preserving alveolar ridge dimensions, accelerating healing, promoting epithelialization, enhancing hemostasis, alleviating pain, reducing trismus, minimizing swelling and mitigating the complications associated with tooth extractions, including mouth opening limitations [2,19,21,24,25,26,27,28,29,30,31,32,35,36,37,38,39,46,51,52,53,54,59,60]. The results presented the use of A-PRF as beneficial, even though A-PRF requires additional steps and machinery, prolonging the overall surgery time and requiring blood donation from the patient.

Regarding the combination of A-PRF with other bio-materials, 13 out of 36 analyzed randomized controlled trials used it in the research. It was most popular in works examining the improvement of new bone formation in conjunction with bone grafting materials [20,25,27,32,33,34,35,36,37,38,39,44,45,57,58]. The evidence of combining A-PRF with grafting materials seems to provide significantly better results, although not all studies obtained favorable results [37]. The main benefit seems to rely on A-PRF providing the essential growth factors quickly, enhancing the bio-material turnover. In two of the studies [54,57], A-PRF also seemed suitable in the event of closing perforation in maxillary sinus mucosa. The positive effect of A-PRF in terms of synergy with bio-materials and improvement of surgery outcomes allows for favorable implantation conditions and more optimized resource usage.

A comparison of the results with other systematic reviews on the topic of A-PRF is not currently possible, as to our knowledge, this is the first review to provide a comprehensive overview of the current A-PRF use cases, which are supported by randomized controlled trials. With the growing interest in natural materials and technological advancements, the future use of A-PRF is only going to expand.

Even though the evidence is of mid-to-high quality, the results still have to be interpreted with caution. Most of the studies specified different approaches to the specific surgeries performed. The variety of bio-materials employed, including autografts, xenografts and enamel matrix derivatives, along with the diverse range of postoperative drug regimens and follow-up protocols, make it challenging to predict whether a consistent outcome can be achieved through the typical surgical approach. Although most studies blinded the participants and randomized the cohorts using special programs to achieve the best randomization, there is always some bias included. Moreover, some of the studies included surgeries performed by more than one surgeon, which can alter the results ever so slightly. The focus on the specific parameters examined can also overshadow other variables, which can influence the results. Although no complications were noted, and patients attended the follow-ups in most studies, there is no guarantee that, on a larger scale, the outcome would be the same. Language limitation was also present in this study, as only publications in English were chosen for conducting this systematic review.

## 5. Conclusions

The utilization of A-PRF is broadening rapidly. There is growing evidence that advanced platelet-rich fibrin has a positive effect on the surgical procedures mentioned. The randomized controlled trials presented mostly favorable results, though it must be noted that such results were observed on a smaller scale. The rapid growth of new technologies, mainly implantology, will accompany the wider and further adoption of A-PRF. Due to its biological properties, A-PRF could be considered a reliable addition to the surgical protocols, both alone and as an additive to bio-materials, with the advantages of healing improvement, pain relief, soft tissue management and bone preservation, as well as graft integration. Nevertheless, further well-designed randomized controlled trials are required for each use case. Ideally, these would include larger patient cohorts, additional research personnel blinded to the treatment allocation and long-term follow up periods. This would allow a determination of the long-term clinical implications and recommendations for clinical practice.

## Figures and Tables

**Table 1:** Studies included in the qualitative analysis and extracted data.

No	References	Aim of the Study	Number of Patients	Follow-Up	Results	Complications
1	Al-Barakani et al., 2024 [24]	The clinical effects of A-PRF or resorbable collagen membrane applied in the treatment of type I and II Miller class gingival recessions using the pin hole surgical technique.	18	1, 2, 3, 4, 3 months	The treatment of recession using A-PRF in the pin hole surgical technique proved more effective than the application of resorbable collagen membrane in the pin hole method. In the case of the treatment of recession with the pin hole method and A-PRF simultaneously, a reduction in the level of postoperative pain was observed.	Not specified
2	Alhaj et al., 2018 [25]	Filling the resultant gap after immediate insertion of a mandibular molar implant with A-PRF + autograft mixture or autograft alone and comparing the outcomes.	20	Days 2 and 7; 3 and 6 months	After 6 months, the crystal bone decrease was more pronounced in the control bone, with statistical significance. A-PRF addition decreased swelling. A-PRF promoted faster regeneration.	Not specified
3	Alsahli et al., 2021 [26]	Comparing palatal free gingival graft and A-PRF as a material for patching uncovered implant sites during healing abutment placement and improving keratinization.	15	1, 4, 8 weeks and 6 months	After 2 weeks of healing, the A-PRF group showed statistically significant improvement in keratinized tissue thickness, but the effect decreased over time. A-PRF improved the width of keratinized tissue, but with no significant advantage over free gingival graft. A-PRF was shown to decrease postoperative morbidity in comparison with free gingival graft.	One patient dropped out
4	Angelo et al., 2015 [27]	Maxillary implant placement after piezotome-enhanced subperiosteal tunnel technique with the use of bio-material with/without A-PRF membrane.	82	6–7 months	The study suggests that A-PRF enhances biomechanic bone quality and allows for better and more consistent results with lower patient morbidity compared to traditional methods.	None
5	Bahammam, 2018 [28]	Patching free gingival graft sites with A-PRF and evaluating its impact on the donor site.	24	1, 2, 3, 4 and 8 weeks	A-PRF is an effective bandage for free gingival graft donor site and helps in the early healing stages of soft tissues by promoting epithelialization.	Not specified
6	Barakat et al., 2024 [29]	The clinical effects of A-PRF or connective tissue graft applied in the treatment of gingival black triangle using Han and Takei’s method.	32	1, 3, 6, 9 and 12 months	A-PRF and connective tissue graft had the same results in the interdental papilla treatment using Han and Takei’s method.	Not specified
7	Brahma Prasad Chary et al., 2021 [20]	The evaluation of treatment outcome for early implant placement in sockets preserved using A-PRF at 6 and 8 weeks following atraumatic extraction.	20	6–8 weeks	Better effects were achieved after 8 weeks (higher insertion torque values and predictable bone).	Not specified
8	Brancaccio et al., 2020 [2]	Extraction of four non-adjacent teeth with treatment using four different hemostatic procedures (sutures only, A-PRF+, HEM, L-PRF).	102	2 weeks	HEM, A-PRF and leukocyte-PRF showed advantage over suturing alone. A-PRF presented a statistically significant advantage over HEM and fared best in bleeding reduction. Only L-PRF reduced the risk of incomplete healing compared to suturing alone with statistical significance. Hypertension and diabetes increased the risk of bleeding, and smoking and diabetes promoted delayed healing.	Not specified
9	Castro et al., 2021 [30]	Patching teeth sockets after multiple extractions in the front maxilla region with A-PRF or L-PRF and measuring the alveolar ridge dimension changes.	21	3 months	Both PRF types could not counteract the progressing bone resorption after 3 months and yielded similar results. Both PRFs turned out to be superior in comparison with unassisted teeth sockets.	Not specified
10	Caymaz et al., 2018 [31]	Managing the socket after third molar extraction with the use of A-PRF and L-PRF.	27	-	A-PRF significantly lowered analgesic usage and the Visual Analog Scale compared to L-PRF, mainly in the first three days following surgery. There was no significant difference in terms of swelling and trismus.	Not specified
11	Clark et al., 2018 [32]	Non-traumatic extraction with the use of A-PRF, A-PRF + FDBA, FDBA or blood clot for ridge preservation and further histomorphologic evaluation of the bone formed.	40	3.75 months	The best results were achieved using A-PRF + FDBA. No significant difference between A-PRF and FDBA in terms of ridge dimension preservation was found. A-PRF and A-PRF + FDBA fared significantly better than blood cloth alone. Using A-PRF or A-PRF + FDBA resulted in formation of a denser trabecular structure. A-PRF also demonstrated the highest percentage of vital bone formation.	Not specified
12	Csifó-Nagy et al., 2021 [21]	Treating periodontal intrabony defects with A-PRF or EMD.	18	6 months	In both groups, the FMBS decreased, and FMPS remained the same. In terms of pocket depth, gingival recession, clinical attachment level and bone sounding changes, A-PRF fared similarly to EMD, showing improvements compared to the baseline; thus, it can be concluded that A-PRF behaves as effectively as EMD in the surgical treatment of intrabony periodontal defects.	None
13	Dayashankara Rao et al., 2020 [33]	Performing secondary alveolar bone grafting using iliac bone graft alone or with a mixture of I- and A-PRF.	30	3 and 6 months	The combination of I + A-PRF with iliac bone graft generated better results than using iliac bone graft alone, with good bone volume and lower chances of resorption. The periodontal status, mobility score and pocket depth improved in both groups, with no statistical significance.	The study group had 6.7% graft failure. The control group had 40% graft failure
14	Dragonas et al., 2023 [34]	Comparison of A-PRF and plasma rich in growth factors combined with DBBM during sinus lift augmentation.	15	6 months	The mean percentage of mineralized bone after the healing period was higher in the group with growth factors, but there was no statistical significance in samples without growth factors. Adding A-PRF and PRGF to DBBM did not improve new bone formation in sinus lift. Neither platelet-rich preparation was better than the other in any of the parameters studied.	Not specified
15	Ghonima et al., 2020 [35]	Regeneration of periodontal intrabony defects using BCP only or together with A-PRF.	22	3, 6, 9 months	At 9 months, both groups had a significantly lower plaque index. At 3-, 6- and 9-month baselines, both groups significantly decreased the PD and gained CAL. No statistically significant difference was observed between A-PRF/BCP and BCP/saline groups, although the A-PRF group noted better results in PD reduction and CAL gain.	Not specified
16	Giudice et al., 2019 [36]	Extraction of four non-adjacent teeth with treatment using four different hemostatic procedures (sutures only, A-PRF+, HEM, L-PRF).	40	1 and 2 weeks	A-PRF+ showed statistically significant bleeding reduction 30 min after extraction. In terms of patient preference and wound healing index, all types of plugs were similarly matched, although the L-PRF and A-PRF groups had higher percentage of complete closures compared to suturing and HEM after a 2-week period.	Not specified
17	Hartlev et al., 2020 [37]	Autogenous bone augmentation in future implant sites with additional use of A-PRF or DBBM and collagen membrane and analysis of vital bone formation.	27	6 months	There were no significant differences between the control and test groups regarding vital bone and non-vital bone formation, the amount of blood vessels and soft tissues.	Two biopsies were discarded due to poor quality control group
18	Hartlev et al., 2021 [38]	Mandibular ramus block harvesting and lateral ridge augmentation with coverage of both sites with either A-PRF/resorbable collagen membrane or deproteinized bovine bone/resorbable collagen membrane.	27	1 and 2 weeks	Both groups experienced low postoperative pain. The A-PRF group experienced lower pain perception, although statistically significant difference was only identified on the first postoperative day.	Changed sensation extra orally in the chin region, bone graft dehiscence in the recipient site control group, sensory disturbances at the recipient site
19	Ivanova et al., 2019 [39]	Extraction using A-PRF only or with FDBA and analyzing its effect on vital bone formation and ridge preservation.	60	4 months	There were no significant differences between the use of allograft and A-PRF in terms of vertical bone resorption and vital bone creation. The use of both A-PRF and allograft outperformed the control group.	Not specified
20	Jayadevan et al., 2021 [40]	The evaluation of A-PRF and PRF as a scaffold in the regenerative endodontic treatment of traumatized immature non-vital permanent anterior teeth.	28	13 months	A-PRF yielded higher root dentin thickness than PRF.	Not specified
21	Kalash et al., 2017 [44]	Immediate implant placement and filling of peri-implant gap with xenograft or PRF–xenograft mixture.	18	Days 2, 7 and 14; 3, 6 and 9 months	The A-PRF and xenograft mixture positively affected soft tissue healing and bone regeneration. Improvement in implant stability was noted, with statistically significant difference.	None
22	Lavagen et al., 2021 [45]	The usage of A-PRF in the treatment of alveolar cleft with iliac bone graft. In the study, the authors evaluated the efficiency of using A-PRF by comparing the volumes of newly formed bone after a bone graft combining autogenous iliac crest bone with either PRF or A-PRF.	24	6 months	In groups with A-PRF placement, bone regeneration was more effective.	Not specified
23	Nowak et al., 2021 [46]	The effect of A-PRF application during surgical extraction of third molars on healing and the concentration of C-reactive protein.	60	7 days	A faster decrease in C-reactive protein levels was shown in patients who used A-PRF after third molar extraction. A-PRF accelerated healing and reduced the occurrence of alveolar osteitis.	Not specified
24	Öngöz Dede et al., 2023 [47]	The clinical effects of concentrated growth factor and A-PRF applied together using the CAF technique in the treatment of type I multiple gingival recessions.	16	6 months	Significant improvements were determined in the clinical attachment level, vertical gingival recession, horizontal gingival recession, gingival thickness, width of keratinized gingiva, percentages of the mean and complete root coverage at 6 months in the CAF + A-PRF group. Mean root coverage was the best in the CAF + A-PRF group.	Not specified
25	Pereira et al., 2023 [48]	The effects of A-PRF+ on the healing of upper third molar post-extraction sockets.	16	90 days	There were no clinical differences regarding healing in any control follow-up.	Not specified
26	Praganta et al., 2024 [49]	The application of A-PRF and gelatin dressing in extraction sockets following mandibular wisdom teeth removal and the influence on postoperative pain and swelling.	87	7 days	A-PRF placement in third molar sockets did not reduce postoperative pain and swelling compared to gelatin dressing alone.	Not specified
27	Rachna M et al., 2024 [50]	The effects of application of A-PRF or A-PRF and the eggshell membrane after teeth extraction.	20	3 and 6 months	In the A-PRF and eggshell membrane group, after 3 and 6 months, the bone density in the cone-beam computed tomography scan was higher than in the A-PRF only group.	Not specified
28	Şen DÖ et al., 2024 [51]	The effects of utilizing L-PRF and A-PRF as a palatal bandage following free gingival graft on patients’ morbidity and oral-health-related quality of life.	39	1–7 and 14 days; 1 and 6 months	The control group without growth factors had higher OHIP-14 total scores than the other groups. The PRF groups showed an improvement in the quality of life and took less painkillers.	Not specified
29	Soto-Penaloza et al., 2019 [52]	Apical root resection (3 mm) with or without the use of A-PRF during free-flap closure.	50	7 days	The difference in pain was not significant between the control and test groups. Taking into account the overall improvement in the quality of life in the test group, A-PRF can be considered as a useful addition to endodontic surgical protocol, as it provides a safe and affordable alternative.	Feeling nauseous, discomfort related to prolonged bleeding and bad breath/taste
30	Sousa et al., 2020 [53]	Patching free gingival graft sites with A-PRF clot membranes and evaluating its potential in improving wound healing.	25	3 months	A-PRF membranes improved the healing process (faster decrease in the wound area and epithelialization promotion) with less postoperative pain.	Hemorrhage (control and study groups), necrosis in the control group (day 7)
31	Starzyńska et al., 2021 [19]	Assessment of the influence of A-PRF on selected clinical features following surgical removal of the impacted mandibular third molars.	100	14 days	A-PRF reduced the pain intensity, analgesic intake, trismus, edema, the presence of hematomas and skin warmth.	Not specified
32	Śmieszek-Wilczewska et al., 2024 [54]	Comparing the effectiveness of PRF and the conventional method in oroantral communication repair techniques.	22	14 days	Complete wound healing.	Oroantral communications treated with A-PRF resulted in fewer complications and less pain
33	Tadepalli et al., 2023 [55]	Assessment of leukocyte platelet-rich fibrin and A-PRF in combination with CAF in the treatment of gingival recession.	30	6 months	Statistically significant reduction in mean recession height was observed from baseline to 6 months in the CAF + L-PRF and CAF + A-PRF groups, respectively. The mean root coverage percentage achieved at 6 months was better in the CAF + A-PRF group (81.66 ± 28.21) than in the CAF + L-PRF group.	Not specified
34	Torul et al., 2020 [56]	Mandibular third molar extraction and evaluation of the effect of connective tissue graft and A-PRF on edema, pain and trismus.	75	14 days	The study showed that connective tissue graft and A-PRF did not exert any significant effects on pain, swelling and trismus.	Not specified
35	Trimmel et al., 2021 [57]	Maxillary sinus augmentation from the lateral approach with the use of serum albumin-coated bone allograft combined with A-PRF.	26	3 and 6 months	Serum albumin-coated bone allograft combined with A-PRF is a suitable material for maxillary sinus augmentation, as augmented and pristine bone showed no significant difference both in histo- and micromorphometric parameters.	In three cases (two in the control group, one in the study group), small perforation was detected and fixed with A-PRF membrane
36	Yewale et al., 2021 [58]	Atraumatic tooth extraction and socket preservation with Sybograf plus or Sybograf plus/A-PRF.	20	6 months	The use of A-PRF increased the effectiveness of the bone graft used in preserving vertical and horizontal dimensions. The swelling percentage in the A-PRF group was noticeably decreased, which led to less discomfort for patients. The pain levels remained equal in both study groups.	None
37	Yüce and Kömerik, 2019 [59]	Managing alveolar osteitis after third molar extraction using A-PRF.	40	1, 3, 7 and 15 days; 1, 2 and 3 months	The use of A-PRF compared to the control group lowered the Visual Analog Scale pain score, which resulted in less analgesics taken. Statistical epithelial healing rates were faster in the A-PRF group. The gray level pixel values comparison showed improved hard tissue healing in the A-PRF group.	Not specified
38	Zahid and Nadershah, 2019 [60]	Assessing the impact on third molar extraction with the use of A-PRF as a regenerative bio-material.	10	1 and 3 months	A-PRF decreased postoperative pain and swelling. A-PRF provided slight but not significant advantages in terms of probing depth reduction, recession coverage and clinical attachment level gain compared to control.	Not specified

A-PRF—advanced platelet-rich fibrin; BCP—biphasic calcium phosphate; CAF—coronally advanced flap; CAL—clinical attachment level; DBBM—deproteinized bovine bone mineral; EMD—enamel matrix derivative; FDBA—freeze-dried bone allografts; FMBS—full mouth bleeding score; HEM—hemostatic plug; L-PRF—leukocyte platelet-rich fibrin; OHIP-14—Oral Health Impact Profile.

## Data Availability

The original contributions presented in the study are included in the article, further inquiries can be directed to the corresponding author.

## Abbreviations

AFG	autologous fibrin glue
A-PRF	advanced platelet-rich fibrin
A-PRF+	advanced platelet-rich fibrin plus
BCP	biphasic calcium phosphate
BMPs	bone morphogenic factors
BMP-2	bone morphogenic factor 2
Ca^2+^	calcium ion
CAL	clinical attachment level
CBCT	cone-beam computed tomography
CGF	concentrated growth factor
CRC	complete root coverage
CRP	C-reactive protein
CTG	connective tissue graft
DBBM	demineralized bovine bone mineral
EMD	enamel matrix derivative
FDBA	freeze-dried bone allografts
FGF	fibroblast growth factor
FGG	free gingival graft
FMBS	full mouth bleeding score
GBT	gingival black triangle
GR	gingival recession
GT	gingival thickness
HEM	hemostatic plug
HGR	horizontal gingival recession
IL-1β	interleukin 1β
IL-4	interleukin IL-4
IL-6	interleukin IL-6
I-PRF	injectable platelet-rich fibrin
ISQ	implant stability quotient
KGW	width of keratinized gingiva
L-PRF	leukocyte–platelet-rich fibrin
micro-CT	micro-computed tomography
MMPs	matrix metalloproteinases
MMP-9	matrix metalloproteinases 9
MRC	percentages of the mean
NSAID	non-steroidal anti-inflammatory drugs
OHIP-14	Oral Health Impact Profile
PD	pocket depth
PDGF	platelet-derived growth factor
PeSPTT	piezotome-enhanced subperiosteal tunnel technique
PICO	population, intervention, comparison, outcome
PRGF	plasma rich in growth factors
PRISMA	Preferred Reporting Items for Systematic Reviews and Meta-Analyses
PROSPERO	Prospective Register of Systematic Reviews
PRF	platelet-rich fibrin
PRGF	plasma rich in growth factors
PRP	platelet-rich plasma
PST	pin hole surgical technique
RCM	resorbable collagen membrane
RCTs	randomized clinical trials
RET	regenerative endodontic treatment
SACBA	serum albumin-coated bone allograft
T-PRF	titanium platelet-rich fibrin
TGF	transforming growth factor
TNFα	tumor necrosis factor α
TGFα	tumor necrosis factor α
TGFβ	tumor necrosis factor β
VAS	Visual Analog Scale
VEGF	vascular-endothelial growth factor
VGR	vertical gingival recession

## References

[1] Dohan D.M., Choukroun J., Diss A., Dohan S.L., Dohan A.J.J., Mouhyi J., Gogly B. (2006). Platelet-rich fibrin (PRF): A secondgeneration platelet concentrate. Part I: Technological concepts and evolution. Oral Surg. Oral Med. Oral Pathol. Oral Radiol. Endod..

[2] Brancaccio Y., Antonelli A., Barone S., Bennardo F., Fortunato L., Giudice A. (2021). Evaluation of local hemostatic efficacy after dental extractions in patients taking antiplatelet drugs: A randomized clinical trial. Clin. Oral Investig..

[3] Choukroun J., Diss A., Simonpieri A., Girard M.O., Schoeffler C., Dohan S.L., Dohan A.J., Mouhyi J., Dohan D.M. (2006). Platelet-rich fibrin (PRF): A second-generation platelet concentrate. Part IV: Clinical effects on tissue healing. Oral Surg. Oral Med. Oral Pathol. Oral Radiol. Endod..

[4] Choukroun J., Diss A., Simonpieri A., Girard M.O., Schoeffler C., Dohan S.L., Dohan A.J., Mouhyi J., Dohan D.M. (2006). Platelet-rich fibrin (PRF): A second-generation platelet concentrate. Part V: Histologic evaluations of PRF effects on bone allograft maturation in sinus lift. Oral Surg. Oral Med. Oral Pathol. Oral Radiol. Endod..

[5] Ghanaati S., Booms P., Orlowska A., Kubesch A., Lorenz J., Rutkowski J., Landes C., Sader R., Kirkpatrick C., Choukroun J. (2014). Advanced platelet-rich fibrin: A new concept for cell-based tissue engineering by means of inflammatory cells. J. Oral Implantol..

[6] Choukroun J. (2014). Advanced PRF, and i-PRF: Platalet concentrates or blood concentrates?. J. Periodont. Med. Clin. Pract..

[7] Upadhayaya V., Arora A., Goyal A. (2017). Bioactive Platelet Aggregates: Prp, Prgf, Prf, Cgf And Sticky Bone. IOSR J. Dent. Med. Sci..

[8] Shirbhate U., Bajaj P. (2022). Third-Generation Platelet Concentrates in Periodontal Regeneration: Gaining Ground in the Field of Regeneration. Cureus.

[9] Illmilda, Asrianti D., Margono A., Julianto I., Wardoyo M.P. (2019). Advanced Platelet Rich Fibrin (A-PRF) supplemented culture medium for human dental pulp stem cell proliferation. J. Int. Dent. Med. Res..

[10] Kobayashi E., Flückiger L., Fujioka-Kobayashi M., Sawada K., Sculean A., Schaller B., Miron R.J. (2016). Comparative release of growth factors from PRP, PRF, and advanced-PRF. Clin. Oral Investig..

[11] Dohan Ehrenfest D.M., Diss A., Odin G., Doglioli P., Hippolyte M.P., Charrier J.B. (2009). In vitro effects of Choukroun’s PRF (platelet-rich fibrin) on human gingival fibroblasts, dermal prekeratinocytes, preadipocytes, and maxillofacial osteoblasts in primary cultures. Oral Surg. Oral Med. Oral Pathol. Oral Radiol. Endod..

[12] Gupta A.K., Cole J., Deutsch D.P., Everts P.A., Niedbalski R.P., Panchaprateep R., Rinaldi F., Rose P.T., Sinclair R., Vogel J.E. (2019). Platelet-Rich Plasma as a Treatment for Androgenetic Alopecia. Dermatol. Surg..

[13] Everts P.A.M., Knape J.T., Weibrich G., Schönberger J.P., Hoffmann J., Overdevest E.P., Box H.A., van Zundert A. (2006). Platelet-rich plasma and platelet gel: A review. J. Extracorpor. Technol..

[14] Kargarpour Z., Nasirzade J., Panahipour L., Mitulović G., Miron R.J., Gruber R. (2021). Platelet-Rich Fibrin Increases BMP2 Expression in Oral Fibroblasts via Activation of TGF-β Signaling. Int. J. Mol. Sci..

[15] Steller D., Herbst N., Pries R., Juhl D., Hakim S.G. (2019). Impact of incubation method on the release of growth factors in non-Ca2+-activated PRP, Ca2+-activated PRP, PRF and A-PRF. J. Craniomaxillofac. Surg..

[16] Miron R.J., Fujioka-Kobayashi M., Sculean A., Zhang Y. (2024). Optimization of platelet-rich fibrin. Periodontology 2000.

[17] Miron R.J. (2021). Understand Platelet Rich Fibrin.

[18] Machut K., Zoltowska A., Pawlowska E., Derwich M. (2021). Plasma Rich in Growth Factors in the Treatment of Endodontic Periapical Lesions in Adult Patients: Case Reports. Int. J. Mol. Sci..

[19] Starzyńska A., Kaczoruk-Wieremczuk M., Lopez M.A., Passarelli P.C., Adamska P. (2021). The Growth Factors in Advanced Platelet-Rich Fibrin (A-PRF) Reduce Postoperative Complications after Mandibular Third Molar Odontectomy. Int. J. Environ. Res. Public Health.

[20] Brahma Prasad Chary N.O., Raju M.S., Suresh Sajjan M.C., Gottumukkala S.N., Manyam R. (2021). Comparison of quality of bone and insertion torque values of early implants placed at 6 and 8 weeks in sockets preserved with advanced platelet-rich fibrin: A randomized controlled trial. J. Indian Prosthodont. Soc..

[21] Csifó-Nagy B.K., Sólyom E., Bognár V.L., Nevelits A., Dőri F. (2021). Efficacy of a new-generation platelet-rich fibrin in the treatment of periodontal intrabony defects: A randomized clinical trial. BMC Oral Health.

[22] Liu Y.H., To M., Okudera T., Wada-Takahashi S., Takahashi S.S., Su C.Y., Matsuo M. (2022). Advanced platelet-rich fibrin (A-PRF) has an impact on the initial healing of gingival regeneration after tooth extraction. J. Oral Biosci..

[23] Bao M., Du G., Zhang Y., Ma P., Cao Y., Li C. (2021). Application of Platelet-Rich Fibrin Derivatives for Mandibular Third Molar Extraction Related Post-Operative Sequelae: A Systematic Review and Network Meta-Analysis. J. Oral Maxillofac. Surg..

[24] Al-Barakani M.S., Al-Kadasi B., Al-Hajri M., Elayah S.A. (2024). A comparative study of the effects of advanced platelet-rich fibrin and resorbable collagen membrane in the treatment of gingival recession: A split-mouth, randomized clinical trial. Head Face Med..

[25] Alhaj F., Shokry M., Attia N. (2018). The efficiency of using advanced platelet rich fibrin–Autogenous bone graft mixture around immediately placed dental implants in mandibular molar region: (Randomized controlled clinical trial). Egypt. Dent. J..

[26] Alsahli J., Kasem T., Alkhouli M. (2021). Evaluation of Apically Positioned flap with A_PRF Vs. Free Gingival Grafts to Enhance the Keratinized Tissue Around Dental Implants: A Randomized Controlled Clinical Split Mouth Trial. Int. J. Dent. Oral Sci..

[27] Angelo T., Marcel W., Andreas K., Izabela S. (2015). Biomechanical Stability of Dental Implants in Augmented Maxillary Sites: Results of a Randomized Clinical Study with Four Different Biomaterials and PRF and a Biological View on Guided Bone Regeneration. BioMed Res. Int..

[28] Bahammam M.A. (2018). Effect of platelet-rich fibrin palatal bandage on pain scores and wound healing after free gingival graft: A randomized controlled clinical trial. Clin. Oral Investig..

[29] Barakat S.O., Tawfik O.K., Kholy S.E., ElNahass H. (2024). Evaluation of advanced platelet-rich fibrin compared to subepithelial connective tissue graft in the surgical management of interdental papilla recession: A randomized controlled trial. Clin. Oral Investig..

[30] Castro A.B., Van Dessel J., Temmerman A., Jacobs R., Quirynen M. (2021). Effect of different platelet-rich fibrin matrices for ridge preservation in multiple tooth extractions: A split-mouth randomized controlled clinical trial. J. Clin. Periodontol..

[31] Caymaz M.G., Uyanik L.O. (2019). Comparison of the effect of advanced platelet-rich fibrin and leukocyte- and platelet-rich fibrin on outcomes after removal of impacted mandibular third molar: A randomized split-mouth study. Niger. J. Clin. Pract..

[32] Clark D., Rajendran Y., Paydar S., Ho S., Cox D., Ryder M., Dollard J., Kao R.T. (2018). Advanced platelet-rich fibrin and freeze-dried bone allograft for ridge preservation: A randomized controlled clinical trial. J. Periodontol..

[33] Dayashankara Rao J.K., Bhatnagar A., Pandey R., Arya V., Arora G., Kumar J., Bootwala F., Devi W.N. (2021). A comparative evaluation of iliac crest bone graft with and without injectable and advanced platelet rich fibrin in secondary alveolar bone grafting for cleft alveolus in unilateral cleft lip and palate patients: A randomized prospective study. J. Stomatol. Oral Maxillofac. Surg..

[34] Dragonas P., Prasad H.S., Yu Q., Mayer E.T., Fidel P.L. (2023). Bone Regeneration in Maxillary Sinus Augmentation Using Advanced Platelet-Rich Fibrin (A-PRF) and Plasma Rich in Growth Factors (PRGF): A Pilot Randomized Controlled Trial. Int. J. Periodontics Restor. Dent..

[35] Ghonima J., El Rashidy M., Kotry G., Abdelrahman H. (2020). The efficacy of combining advanced platelet-rich fibrin to biphasic alloplast in management of intrabony defects (randomized controlled clinical trial). Alex. Dent. J..

[36] Giudice A., Esposito M., Bennardo F., Brancaccio Y., Buti J., Fortunato L. (2019). Dental extractions for patients on oral antiplatelet: A within-person randomised controlled trial comparing haemostatic plugs, advanced-platelet-rich fibrin (A-PRF+) plugs, leukocyte- and platelet-rich fibrin (L-PRF) plugs and suturing alone. Int. J. Oral Implantol..

[37] Hartlev J., Erik Nørholt S., Spin-Neto R., Kraft D., Schou S., Isidor F. (2020). Histology of augmented autogenous bone covered by a platelet-rich fibrin membrane or deproteinized bovine bone mineral and a collagen membrane: A pilot randomized controlled trial. Clin. Oral Implant. Res..

[38] Hartlev J., Nørholt S.E., Schou S., Isidor F. (2021). Pain after mandibular ramus block harvesting and lateral ridge augmentation with and without involvement of platelet-rich fibrin: A randomized controlled trial. Int. J. Oral Maxillofac. Surg..

[39] Ivanova V., Chenchev I., Zlatev S., Kanazirski N. (2019). Dimensional Ridge Alterations and Histomorphometric Analysis Following Socket Preservation with PRF or Allograft. Randomized Controlled Clinical Study. J. IMAB–Annu. Proc. Sci. Pap..

[40] Jayadevan V., Gehlot P.M., Manjunath V., Madhunapantula S.V., Lakshmikanth J.S. (2021). A comparative evaluation of Advanced Platelet-Rich Fibrin (A-PRF) and Platelet-Rich Fibrin (PRF) as a Scaffold in Regenerative Endodontic Treatment of Traumatized Immature Non-vital permanent anterior teeth: A Prospective clinical study. J. Clin. Exp. Dent..

[41] Liberati A., Altman D.G., Tetzlaff J., Mulrow C., Gøtzsche P.C., Ioannidis J.P.A., Clarke M., Devereaux P.J., Kleijnen J., Moher D. (2009). The PRISMA statement for reporting systematic reviews and meta-analyses of studies that evaluate health care interventions: Explanation and elaboration. Ann. Intern. Med..

[42] Higgins J.P.T., Sterne J.A.C., Savović J., Page M.J., Hróbjartsson A., Boutron I., Reeves B., Eldridge S., Chandler J., McKenzie J., Boutron I., Welch V. (2016). A revised tool for assessing risk of bias in randomized trials. Cochrane Methods: Cochrane Database of Systematic Reviews.

[43] Sterne J.A.C., Savović J., Page M.J., Elbers R.G., Blencowe N.S., Boutron I., Cates C.J., Cheng H.Y., Corbett M.S., Eldridge S.M. (2019). RoB 2: A revised tool for assessing risk of bias in randomised trials. BMJ.

[44] Kalash S., Aboelsaad N., Shokry M., Choukroun J. (2017). The efficiency of using advanced PRF-xenograft mixture around immediate implants in the esthetic zone: A randomized controlled clinical trial. J. Osseointegr..

[45] Lavagen N., Nokovitch L., Algrin A., Dakpe S., Testelin S., Devauchelle B., Gbaguidi C. (2021). Efficiency of advanced-PRF usage in the treatment of alveolar cleft with iliac bone graft: A retrospective study. J. Craniomaxillofac. Surg..

[46] Nowak J.M., Surma S., Romańczyk M., Wojtowicz A., Filipiak K.J., Czerniuk M.R. (2021). Assessment of the Effect of A-PRF Application during the Surgical Extraction of Third Molars on Healing and the Concentration of C-Reactive Protein. Pharmaceutics.

[47] Öngöz Dede F., Bozkurt Doğan Ş., Çelen K., Çelen S., Deveci E.T., Seyhan Cezairli N. (2023). Comparison of the clinical efficacy of concentrated growth factor and advanced platelet-rich fibrin in the treatment of type I multiple gingival recessions: A controlled randomized clinical trial. Clin. Oral Investig..

[48] Pereira D.A., Mendes P.G.J., Prisinoto N.R., de Rezende Barbosa G.L., Soares P.B.F., de Oliveira G.J.P.L. (2023). Advanced platelet-rich-fibrin (A-PRF +) has no additional effect on the healing of post-extraction sockets of upper third molars. A split mouth randomized clinical trial. Oral Maxillofac. Surg..

[49] Praganta J., De Silva H., De Silva R., Tong D.C., Thomson W.M. (2024). Effect of Advanced Platelet-Rich Fibrin (A-PRF) on Postoperative Level of Pain and Swelling Following Third Molar Surgery. J. Oral Maxillofac. Surg..

[50] Rachna M., Nandita S., Rashmi K.S., Vidya G.B., Srikant N., Ananya J., Dharnappa P. (2024). Eggshell membrane as a regenerative material in alveolar bone grafting in combination with advanced platelet rich fibrin. Clin. Ter..

[51] Şen D.Ö., Şengül B.I., Yarkaç F.U., Öncü E. (2024). Impact of platelet-rich fibrin derivatives on patient morbidity and quality of life in palatal donor sites following free gingival graft surgery: A randomized clinical trial. Clin. Oral Investig..

[52] Soto-Peñaloza D., Peñarrocha-Diago M., Cervera-Ballester J., Peñarrocha-Diago M., Tarazona-Alvarez B., Peñarrocha-Oltra D. (2020). Pain and quality of life after endodontic surgery with or without advanced platelet-rich fibrin membrane application: A randomized clinical trial. Clin. Oral Investig..

[53] Sousa F., Machado V., Botelho J., Proença L., Mendes J.J., Alves R. (2020). Effect of A-PRF Application on Palatal Wound Healing after Free Gingival Graft Harvesting: A Prospective Randomized Study. Eur. J. Dent..

[54] Śmieszek-Wilczewska J., Balicz A., Morawiec T., Al-Maawi S., Heselich A., Sader R., Rutkowski J.L., Mourão C.F., Ghanaati S. (2024). Effectiveness of Oroantral Communication Closure Using Solid Platelet-Rich Fibrin Compared to a Conventional Treatment Approach: A Randomized Clinical Trial. J. Oral Implantol..

[55] Tadepalli A., Chekurthi S., Kavassery Balasubramanian S., Parthasarathy H., Ponnaiyan D. (2022). Comparative Evaluation of Clinical Efficacy of Leukocyte-Rich Platelet-Rich Fibrin with Advanced Platelet-Rich Fibrin in Management of Gingival Recession Defects: A Randomized Controlled Trial. Med. Princ. Pract..

[56] Torul D., Omezli M.M., Kahveci K. (2020). Evaluation of the effects of concentrated growth factors or advanced platelet rich-fibrin on postoperative pain, edema, and trismus following lower third molar removal: A randomized controlled clinical trial. J. Stomatol. Oral Maxillofac. Surg..

[57] Trimmel B., Gyulai-Gaál S., Kivovics M., Jákob N.P., Hegedűs C., Szabó B.T., Dobó-Nagy C., Szabó G. (2021). Evaluation of the Histomorphometric and Micromorphometric Performance of a Serum Albumin-Coated Bone Allograft Combined with A-PRF for Early and Conventional Healing Protocols after Maxillary Sinus Augmentation: A Randomized Clinical Trial. Materials.

[58] Yewale M., Bhat S., Kamath A., Tamrakar A., Patil V., Algal A.S. (2021). Advanced platelet-rich fibrin plus and osseous bone graft for socket preservation and ridge augmentation—A randomized control clinical trial. J. Oral Biol. Craniofac. Res..

[59] Yüce E., Kömerik N. (2019). Potential effects of advanced platelet rich fibrin as a wound-healing accelerator in the management of alveolar osteitis: A randomized clinical trial. Niger. J. Clin. Pract..

[60] Zahid T.M., Nadershah M. (2019). Effect of Advanced Platelet-rich Fibrin on Wound Healing after Third Molar Extraction: A Split-mouth Randomized Double-blind Study. J. Contemp. Dent. Pract..

